# Is waiting for rewards good for you? No association between impulsive choice, psychopathology, and functional outcomes in a large cohort sample

**DOI:** 10.1002/jcv2.12231

**Published:** 2024-04-15

**Authors:** Patricia P. Bado, Giovanni A. Salum, Luis A. Rohde, Ary Gadelha, Pedro M. Pan, Eurípedes C. Miguel, Gail Tripp, Emi Furukawa

**Affiliations:** ^1^ Hospital de Clínicas de Porto Alegre (HCPA) Universidade Federal Do Rio Grande Do Sul (UFRGS) Porto Alegre Brazil; ^2^ Pontifícia Universidade Católica do Rio de Janeiro (PUC‐Rio) Rio de Janeiro Brazil; ^3^ National Institute of Developmental Psychiatry for Children and Adolescents & National Center for Innovation and Research in Mental Health São Paulo Brazil; ^4^ ADHD Outpatient Program & Developmental Psychiatry Program Hospital de Clínicas de Porto Alegre Federal University of Rio Grande Do Sul Porto Alegre Brazil; ^5^ Medical Council UniEduK São Paulo Brazil; ^6^ Universidade Federal de São Paulo (UNIFESP) São Paulo Brazil; ^7^ Laboratório de Neurociências Integrativas (Linc) Universidade Federal de São Paulo (UNIFESP) São Paulo Brazil; ^8^ Universidade de São Paulo (USP) São Paulo Brazil; ^9^ Okinawa Institute of Science and Technology (OIST) Graduate University Onna‐son Okinawa Japan

**Keywords:** ADHD, choice delay task, life outcomes, longitudinal, psychopathology

## Abstract

**Background:**

A stronger preference for immediate rewards has been reported in individuals with ADHD and other disorders. However, the consistency of the associations between this preference and psychiatric conditions as well as functional outcomes have been questioned. Research on its association with longitudinal outcomes is scarce.

**Methods:**

The current study used data on a choice delay task (CDT) from a school‐based cohort of Brazilian children with those at higher risk for psychiatric disorders over‐sampled (*n* = 1917). The sample included typically developing children (*n* = 1379), those with ADHD (*n* = 213), and other disorders. The frequency of the trials where children chose a larger later reward versus a smaller sooner reward was compared for those with ADHD and typically developing children. Cross‐sectionally and longitudinally, the study also evaluated whether children's preference for larger delayed rewards at baseline predicted the presence of psychiatric disorders and functional life outcomes (academic performance, alcohol use, early pregnancy, criminal conviction, BMI).

**Results:**

Children with ADHD and their typically developing peers performed similarly on the CDT. Their baseline task performance was not related to psychiatric conditions or life outcomes.

**Conclusions:**

The current results raise questions regarding the use of the CDT with diverse populations and whether a preference for larger delayed rewards is predictive of positive long‐term outcomes as widely assumed.

**Key points**

- Impulsive choice is assumed to be maladaptive, although longitudinal evidence for its negative impacts on psychopathology and functional outcomes is limited.
- In a large community cohort sample, preference for smaller sooner versus larger later rewards did not differ for children with and without ADHD.
- Preference for larger later rewards did not predict longitudinal psychiatric or functional outcomes.
- Increased sample and methodological diversity is needed in evaluating behavioral preference for immediate rewards in ADHD and other disorders.

## INTRODUCTION

Altered reinforcement sensitivity has been hypothesized to account for a range of maladaptive behaviors across multiple psychiatric disorders (Amlung et al., 2019), including substance use (Bickel et al., 2014; Reynolds, 2006), obesity (Weller et al., 2008), and especially attention‐deficit/hyperactivity disorder (ADHD) (Sagvolden et al., 1998; Tripp & Wickens, 2008). Within the field of ADHD, sensitivity to delayed reward has been studied extensively. Research shows that individuals with ADHD demonstrate a stronger preference for small immediate over larger delayed rewards than typically developing (TD) individuals (e.g., Furukawa et al., 2022; Sonuga‐Barke, 2002).

This preference for immediacy has been widely studied using the simple choice paradigm (SCP) (Marx et al., 2021). It employs a two‐alternative forced choice task, in which individuals repeatedly choose between two available, fixed options that differ in the size of the reward and the time to reward delivery. The choice is made at the beginning of each trial, after which individuals experience the actual delay, typically in the seconds‐range. The temporal discounting paradigm (TDP) also measures temporal reward preference through individuals' choices between two varied options of different delay length and reward size. The choices are usually hypothetical and of relatively large amounts of money (tens and hundreds of dollars) and time (days, weeks, and years). The SCP is more commonly used in studies of ADHD and with children. The TDP is also used in ADHD research, but more widely in studies of other disorders and with typically developing adolescent and adult populations.

A number of studies using SCP report that individuals with ADHD are less likely to choose larger later (LL) than smaller sooner (SS) rewards, compared to their TD peers (Marx et al., 2021). However, this effect is not universal, with other studies reporting no between‐group differences (Bidwell et al., 2007; Solanto et al., 2007; Wåhlstedt, 2009). There is also a marked variability in the degree of preference (i.e., percentage of trials where children choose the LL reward) within the ADHD and control groups across studies (Marx et al., 2021). The most recent meta‐analysis on these SCP studies indicates a significant publication bias (Marx et al., 2021), with many small studies with 20 participants or less per group (Dalen et al., 2004; e.g., Marx et al., 2010; Sonuga‐Barke et al., 1992; Vloet et al., 2010) contributing to the average effect.

The robustness of group differences on the temporal reward preference may also depend on sample selection. Of the SCP studies included in the meta‐analysis by (Marx et al., 2021), most used clinical samples (individuals recruited in clinic settings) and carefully selected control groups. In their studies with community samples, Bidwell et. (2007) and Wåhlstedt et al. (2009) did not find group differences between those meeting the DSM criteria for ADHD versus not. Moreover, most of the reviewed studies were conducted in higher‐income, North‐American and European countries, with the exception of three studies undertaken in China (Yang et al., 2011; Yu et al., 2018; Zhu et al., 2015). Outside the field of ADHD, research suggests the socioeconomic levels of the study population affect preference for immediacy (Ho et al., 2022; Ruggeri et al., 2022). Using the temporal discounting paradigm, these studies show individuals in lower‐income countries discount the value of a reward more steeply when its availability is delayed. Thus, the generalizability of the SCP findings in ADHD, especially to community samples of socioeconomically and culturally diverse populations, is uncertain. If a population demonstrates a stronger preference for immediate reward, the difference between individuals with and without ADHD may be smaller.

The ability to wait for rewards has been assumed to be adaptive (Jarmolowicz et al., 2014; Ludwig et al., 2019; Mischel, 2014; Story et al., 2014). However, the longitudinal functional effects are not well characterized. In an early study, Mischel et al. (1989) showed that children who wait for delayed rewards attain higher academic aptitude and self‐control scores, although this finding has not been consistently replicated (Michaelson & Munakata, 2020; Watts et al., 2018). Steeper discounting of delayed reward has been linked to cross‐sectional substance use risk (Anokhin et al., 2011; Strickland et al., 2021), weight gain (Weller et al., 2008), lower academic performance (Mischel et al., 1989), and criminal behavior (Lee et al., 2017). However, little longitudinal evidence is available to indicate that waiting for delayed rewards is related to these risks or negative outcomes (Ho et al., 2022; Isen et al., 2014). We were unable to identify any studies that evaluated the relationship between performance on the SCP and longer‐term functional outcomes. Using a TDP, De Rosa and colleagues (2023) recently demonstrated longitudinal associations between preference for immediately available reward and multiple psychiatric conditions, including ADHD, from childhood through adolescence. They also showed persistent and consistent effects of intellectual ability and income level on such preferences. The relationship between performance on the task and functional outcomes was not examined. To our knowledge, longitudinal TDP studies examining functional outcomes are limited to those reporting modest effects on mortality risk in older adults (Boyle et al., 2013), postpartum smoking relapse (Yoon et al., 2007), and adolescence cigarette smoking (Audrain‐McGovern et al., 2009). A recent meta‐analysis (Lu et al., 2023) reported limited evidence of association between risky or unhealthy behaviors and age‐related changes in discounting rates. There is a clear need to clarify the longitudinal, functional significance of altered sensitivity to reward delay, including among diverse populations.

The current study uses data from the Brazilian High‐Risk Cohort for Psychiatry Disorders (BHRC) (Salum et al., 2015). A choice delay task (CDT), the most widely used SCP, was administered during the baseline assessment, to determine if children's performance on this task is associated with psychiatric and functional risks across the lifespan. Here, we compare the baseline CDT performance of children with and without ADHD in this large, community, lower‐income Brazilian sample. We also evaluate whether CDT performance at baseline predicts cross‐sectional and longitudinal psychiatric risk (ADHD, behavior disorders, emotional disorders, or any psychiatric disorder) and long‐term life outcomes (academic performance, alcohol use, BMI, criminal conviction, or pregnancy) in adolescence and young adulthood. We hypothesize that children meeting the DSM ADHD criteria will make fewer larger later reward choices, compared to typically developing children (those not meeting criteria for ADHD or another psychiatric disorder). We also predict children who make fewer delayed choices will be more likely to meet the criteria for a psychiatric disorder and experience more negative life outcomes.

## METHODS

This study was approved by the ethics committees of the University of São Paulo, the Federal University of Rio Grande do Sul, and other local institutions that took part in the data collection. All participants and their caregivers provided informed written consent.

### Participants and study design

The Brazilian High‐Risk Cohort for Psychiatry Disorders (BHRC) is a school‐based cohort from the cities of São Paulo and Porto Alegre. Children with a higher susceptibility for psychiatric disorders were over‐sampled based on the Family History Screen (FHS) (Weissman et al., 2000) interview conducted with families of children registered in 57 elementary schools. The current study uses data from this cohort. Sample characteristics and data collection procedures relevant to the current study are described below. For detailed information about the cohort, see Salum et al. (2015).

Altogether 2511 children/adolescents from the BHRC and their parents were assessed at baseline. Of those, 2131 children completed a version of the CDT during the baseline assessment. Data from 214 participants were excluded from data analysis due to an IQ below 70 (33), missing data for IQ (150), technical problems with the task (16), the child's inability to understand the task instructions (4), or non‐optimal environment for task administration (11). The final dataset included 1917 participants (male *n* = 1046 (55%), age mean = 10.22 years, range = 5.83–14.17) (Table 1). Performance on the CDT did not differ for those included (mean LL count = 5.98) and excluded (mean LL count = 6.03; *t* = 0.43, *p* = 0.67). At the 3‐year follow‐up assessment, 1569 participants (male *n* = 871 (55%), age mean = 13.49, range = 9.20–17.60) took part in the data collection (82% retention rate), and 1386 participants (male *n* = 747 (54%), age mean = 18.21, range = 13.45–22.83) at the 6‐year follow‐up (72% retention rate), with no significant effect of psychiatric diagnosis or the baseline CDT performance (LL count) on the likelihood of dropping out from the study. The number of children meeting DSM criteria for ADHD were 213 (11.11%), 73 (4.65%), and 40 (2.80%) at baseline, and at the three‐ and 6‐year follow‐ups, respectively (see Table 1 for the rates of other disorders). Socioeconomic status of the participants at baseline ranged from 2 to 37 (mean = 18.26) as measured by Brazilian Association of Research Companies (ABEP) classification. The 2010 revision of the classification indicates ABEP scores of 0–13 correspond to classes D and E (low), 14–22 to class C (low middle), and 23–46 to classes B and A (upper middle and high income) in Brazil; 11.4% of participants were low, 71.8% low middle, and 16.7% middle and high income.

**TABLE 1:** Participant characteristics and CDT task performance.

	Participant *n* (% total)	Age M (sd; range)	Male sex *n* (%)	SES M (sd; range)	IQ (M (sd; range)	LL^b^ count M (sd; range)	LL % M (sd; range)
Disorder presence^a^							
Baseline	**1917**	10.22 (1.92; 5.83–14.17)	1046 (54.56)	18.26 (4.47; 2–37)	100.62 (14.40; 70.37–151.46)	5.98 (3.67; 0–15)	39.84 (24.47; 0–100)
ADHD	213 (11.11)	10.07 (1.76; 6.79–13.97)	130 (61.03)	17.98 (4.07; 6–31)	97.51 (13.98; 71.24–137.42)^c^	6.44 (3.86; 0–15)	42.91 (25.76; 0–100)
Behavioral	135 (7.04)	10.16 (1.83; 6.81–13.97)	84 (62.22)	17.19 (4.13; 7–30)	98.59 (13.75; 71.24–133.56)	5.85 (3.46; 0–15)	39.01 (23.09; 0–100)
Emotional	267 (13.93)	10.35 (1.87; 6.61–13.90)	133 (49.81)	17.86 (3.97; 7–29)	99.06 (14.32; 70.37–138.35)	5.98 (3.60; 0–15)	39.85 (24.01; 0–100)
Any	518 (27.02)	10.21 (1.84; 6.61–13.97)	290 (55.98)	17.90 (4.13; 6–31)	98.97 (14.20; 70.37–138.35)	6.11 (3.65; 0–15)	40.75 (24.31; 0–100)
TD (no disorder)	1379 (71.93)	10.20 (1.94; 5.83–14.17)	744 (53.95)	18.41 (4.59; 2–37)	101.28 (14.45; 70.77–151.46)	5.94 (3.68; 0–15)	39.56 (24.54; 0–100)
3 year follow‐up	**1569**	13.49 (1.92; 9.20–17.60)	871 (55.51)				
ADHD	73 (4.65)	12.93 (1.78; 10.18–17.31)	49 (67.12)				
Behavioral	86 (5.48)	13.62 (1.85; 10.26–17.31)	52 (60.46)				
Emotional	266 (16.95)	13.59 (1.93; 9.89–17.19)	116 (43.61)				
Any	360 (22.94)	15.53 (1.92; 9.89–17.31)	179 (49.72)				
TD (no disorder)	1191 (75.91)	13.48 (1.92; 9.21–17.60)	679 (57.01)				
6 year follow‐up	**1386**	18.21 (2.00; 13.45–22.83)	747 (53.90)				
ADHD	40 (2.89)	17.97 (2.08; 14.31–21.80)	27 (67.50)				
Behavioral	48 (3.46)	18.76 (1.88; 14.37–21.88)	26 (54.17)				
Emotional	334 (24.10)	18.35 (1.93; 14.49–22.29)	119 (35.63)				
Any	390 (28.14)	18.32 (1.93; 14.31–22.29)	155 (39.74)				
TD (no disorder)	1067 (76.98)	18.16 (2.01; 13.45–22.83	583 (54.64)				

*Note*: Participants' characteristics are presented at baseline, 3 and 6‐year follow‐up. DSM‐IV diagnoses for ADHD, behavior disorders (ODD/CD), emotional disorders (mood and anxiety disorders), and any psychiatric disorder (Any).

Abbreviations: %LL, percentage of choices for a larger later reward; LL count, number of trials children chose a larger later reward; M, mean; SES, socioeconomic status.

^a^ Children meeting the criteria for the DSM‐IV diagnostic criteria, Includes those with other disorders, thus not mutually exclusive.

^b^ Trials on which a child chose a larger later reward.

^c^ Significant difference when compared with TD group (*p* < .001).

### Choice delay task

A 15‐trial version^1^ of the choice delay task (CDT) (Sonuga‐Barke et al., 1992) was administered to children at their school by trained mental health professionals. Children were asked to choose between waiting 2 s to get 1 point or waiting 20 s to get 2 points on 15 separate trials. Children made the choice at the beginning of each trial and then waited to receive the points. They were told that they could exchange acquired points for candies at the end of the CDT. The number of trials on which the child chose the larger delayed reward (LL count) was recorded and used in the analysis.

### Psychopathology and life outcomes

The Development and Well‐Being Assessment (DAWBA) (Goodman et al., 2000) was used to evaluate whether children meet the criteria for DSM‐IV diagnoses. Trained lay interviewers administered the structured interview with caregivers, and all resulting diagnoses were reviewed by psychiatrists (see Salum et al., 2015). This procedure was repeated at the 3‐year follow up. At the 6‐year follow up, the diagnostic interviews were carried out with caregivers for cohort participants below 18 years of age, and with participants themselves if older than 18 years. The current study used the following variables: the presence of ADHD, behavior disorders (ODD/CD), emotional disorders (mood and anxiety disorders), and of any psychiatric disorder (Table 1). To further examine the relationship between ADHD‐related behavior and the LL count at baseline, caregiver ratings on the hyperactivity/inattention scale from Strengths and Difficulties Questionnaire (SDQ) (Goodman et al., 2000) were extracted. Variables available from the BHRC are described at https://osf.io/ktz5h/.

Caregivers and cohort participants themselves were interviewed for other information following the BHRC's study protocol. This included questions on child and family demographics, health history, academic functioning, and significant life events (see Salum et al., 2015). In the current analysis, we used the following variables: academic performance, evaluated for participants below 18 years who were still in school, based on caregiver ratings on eight academic subjects on a five‐point scale, averaged and z‐transformed. The cohort participants themselves were interviewed by a psychologist, without their caregiver present, regarding their alcohol use (never, once or twice, monthly, weekly, or daily/almost daily in the last 12 months); ever becoming pregnant/responsible for a partner's pregnancy (separately for girls and boys); and criminal convictions (ever convicted for a crime). The participants' BMI was calculated based on measurements taken during their data collection visit. These variables were selected based on findings from previous delay gratification and temporal discounting studies.

### Data analysis

Children's sex, age, SES and IQ were used as control variables in examining the ADHD versus TD group differences and associations between the LL count and psychiatric and life outcome variables. Prior to these analyses, we conducted a *t*‐test to examine for sex differences on the LL count, and correlations to examine for associations between LL count and age, SES, and IQ. The significance threshold was set at *p* = 0.001 given the sample size and multiple comparisons.

ANOVA was used to examine the mean difference (adjusted for control variables) in the LL count between ADHD and TD groups. Exploratory analyses also examined mean differences when including only a non‐comorbid, unmedicated ADHD subsample and when separated into three age groups (6–8, 9–11, 12–14 year‐olds). The interaction effect of group and sex on the LL count in a two‐way ANOVA, and the correlation between the LL count and SDQ hyperactivity/inattention scale score was also explored.

Hierarchical logistic regression analyses were conducted to evaluate whether the LL count predicts the diagnosis of ADHD, behavior disorders, emotional disorders, or any disorder both concurrently (baseline) and longitudinally (at 3 and 6‐year‐follow ups). The control variables were entered first in the model, then the LL count. The overall fit, and the contribution of LL count and control variables to each dependent variable were examined separately. Odds ratios (OR) were calculated to evaluate the magnitude of their contributions.

Logistic regression analyses were also conducted to evaluate whether the LL count predicts early pregnancy, criminal conviction, and alcohol use at the 6‐year follow‐up when participants had the opportunity to engage in these behaviors. Hierarchical linear regressions were also conducted to evaluate whether the LL count predicts academic performance, alcohol use, and BMI at the 6‐year follow‐up.

## RESULTS

For the entire sample, there was no sex difference in the LL count (*t* = 1.15, df = 1876.1, *p* = 0.250). Correlations between the LL count and SES (*r* = 0.02, *p* = 0.393), IQ (*r* = 0.03, *p* = 0.119) and age (*r* = 0.06, *p* = 0.014) were small. These variables were still included as control variables in the analyses given their likely contributions to the dependent variables examined.

### ADHD versus TD

No significant difference in the LL count was observed between the ADHD (*n* = 213; LL adjusted mean = 6.45 [43%]) and TD (*n* = 1379; LL adjusted mean = 5.94 [40%]) groups, F (1, 1586) = 3.55, *p* = 0.060.^2^ The result was unchanged when comparing the TD children (*n* = 1379; LL = 5.94 [40%]) with a non‐comorbid, unmedicated ADHD subsample (*n* = 106; LL = 6.72 [45%]), F (1, 1479) = 4.42, *p* = 0.036. There were no significant effects of covariates in either analysis.

There were also no significant ADHD versus TD differences on the LL count when examining the three age groups separately, 6–8 year‐olds (F (1, 487) = 0.03, *p* = 0.867), 9–11 year‐olds (F (1, 761) = 5.24, *p* = 0.867), and 12–14 year‐olds (F (1, 326) = 0.98, *p* = 0.322). In a two‐way ANOVA, no interaction effect of sex and ADHD versus TD group on the LL count was observed, F (1, 1585) = 0.13, *p* = 0.722.^3^ The partial correlation between the LL count and SDQ hyperactivity/inattention scale score was not significant, *r* (1568) = −0.001, *p* = 0.970. See Supplemental Tables S1–S4 for descriptive statistics.

### Psychiatric disorders

**FIGURE 1:**
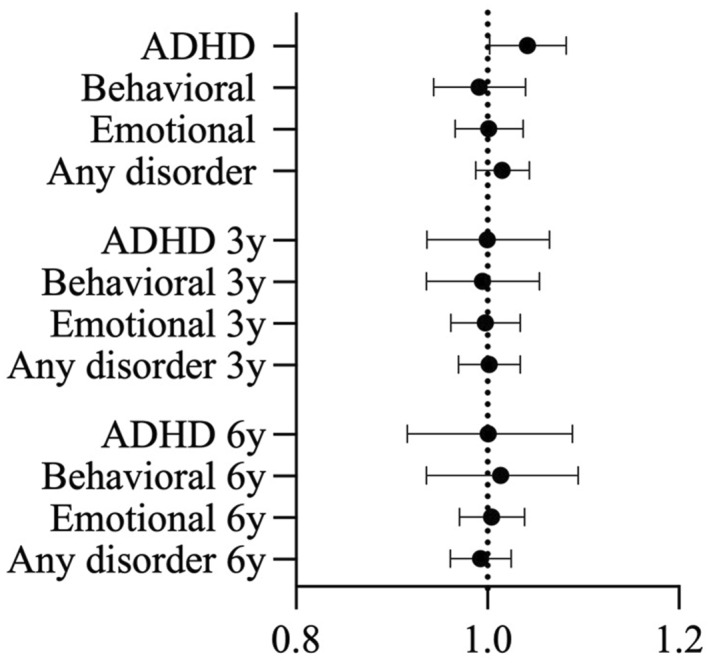
The effect sizes for associations between delayed reward choices and psychopathology. Odds ratios and 95% confidence intervals for children meeting diagnostic criteria versus not, according to their LL counts at baseline. Values greater than one indicate increased risks for psychopathology with greater frequencies of trials in which children chose a larger later reward. 3** **y: 3‐year follow‐up; 6** **y: 6‐year follow‐up.

Overall fit of the logistic regression models was sufficient (Table 2). The LL count did not predict the presence of ADHD, behavior disorders, emotional disorders, or any psychiatric disorder, concurrently or prospectively (Figure 1, Table 2). Results were unchanged when the diagnosis at baseline was included in the regression analyses.

**TABLE 2:** Logistic regression models predicting current and future psychopathology.

	t/z	*p*	B	Chi‐square	df	*p*
Baseline						
ADHD				21.435	5	<0.001
LL count	2.075	0.038	0.041			
IQ	−3.310	0.001	−0.018			
SES	−0.600	0.549	−0.010			
Age	−1.158	0.247	−0.045			
Sex	−1.937	0.053	−0.290			
Behavioral				14.444	5	0.013
LL count	−0.358	0.720	−0.009			
IQ	−1.296	0.195	−0.008			
SES	−2.737	0.006	−0.058			
Age	−0.210	0.834	−0.010			
Sex	−1.970	0.049	−0.365			
Emotional				9.457	5	0.092
LL count	0.073	0.942	0.001			
IQ	−1.699	0.089	−0.008			
SES	−1.278	0.201	−0.019			
Age	1.157	0.248	0.040			
Sex	1.565	0.118	0.208			
Any disorder				14.265	5	0.014
LL count	1.085	0.278	0.015			
IQ	−2.802	0.005	−0.010			
SES	−1.738	0.082	−0.021			
Age	<0.001	1.000	<0.001			
Sex	−0.817	0.414	−0.085			
3‐year follow‐up						
ADHD				13.804	5	0.017
LL count	−0.005	0.996	0.000			
IQ	−2.141	0.032	−0.019			
SES	0.241	0.810	0.007			
Age	−2.126	0.034	−0.140			
Sex	−1.941	0.053	−0.497			
Behavioral				6.166	5	0.290
LL count	−0.182	0.856	−0.005			
IQ	−0.911	0.362	−0.007			
SES	−1.725	0.085	−0.046			
Age	0.932	0.351	0.054			
Sex	−1.055	0.292	−0.240			
Emotional				25.006	5	<0.001
LL count	−0.128	0.898	−0.002			
IQ	−2.135	0.033	−0.010			
SES	0.083	0.934	0.001			
Age	1.468	0.142	0.052			
Sex	4.224	<0.001	0.576			
Any disorder				13.936	5	0.016
LL count	0.095	0.925	0.002			
IQ	−2.325	0.020	−0.010			
SES	−0.557	0.578	−0.008			
Age	1.174	0.241	0.037			
Sex	2.472	0.014	0.299			
6‐year follow‐up						
ADHD				7.571	5	0.182
LL count	0.014	0.989	0.001			
IQ	−1.229	0.219	−0.015			
SES	−1.274	0.203	−0.050			
Age	−0.700	0.484	−0.061			
Sex	−1.728	0.084	−0.601			
Behavioral				4.332	5	0.503
LL count	0.347	0.729	0.014			
IQ	−1.166	0.244	−0.012			
SES	−0.780	0.435	−0.027			
Age	1.445	0.149	0.111			
Sex	−0.154	0.878	−0.045			
Emotional				50.023	5	<0.001
LL count	0.258	0.796	0.004			
IQ	2.201	0.028	0.010			
SES	−0.472	0.637	−0.007			
Age	0.636	0.525	0.021			
Sex	6.555	<0.001	0.843			
Any disorder				35.608	5	<0.001
LL count	−0.440	0.660	−0.007			
IQ	1.783	0.075	0.007			
SES	−0.510	0.610	−0.007			
Age	0.510	0.610	0.016			
Sex	5.566	<0.001	0.671			

*Note*: DSM‐IV diagnoses for ADHD, behavior disorders (ODD/CD), emotional disorders (mood and anxiety disorders), and any psychiatric disorder (Any).

Abbreviation: LL count, number of trials children chose a larger later reward.

The contribution of IQ to ADHD diagnosis was significant. Significant contributions of sex were observed for the presence of an emotional disorder or any disorder. Females were at increased risk for emotional disorders at the 3‐year and 6‐year‐follow ups, and for any psychiatric disorders at the 6‐year‐follow up (Table 2, Figure S1, Supplemental Table S5).

### Life outcomes

Overall fit of the logistic and linear regression models was sufficient (Table 3). The LL count did not predict any of the life outcomes at the 6‐year follow‐up (i.e., academic performance, alcohol use, pregnancy, criminal conviction, BMI; Table 3).

**TABLE 3:** Logistic and linear regression models predicting life outcomes.

	*t*/z	*p*	*B*	F/Chi‐square	df	*p*
Academic performance^a^				7.121	5	<0.001
*n* = 633, M (sd) = 0.002 (0.093), range = −2.92–1.77						
LL count^c^	−0.167	0.868	−0.002			
IQ	4.143	<0.001	0.010			
SES	2.492	0.013	0.021			
Age	0.104	0.917	0.004			
Sex	3.253	0.001	0.238			
Alcohol use frequency^a^				26.28	5	<0.001
*n* = 1161, M (sd) = 1.23 (1.09), range = 0‐4						
LL count	−0.724	0.469	−0.006			
IQ	2.365	0.018	0.005			
SES	0.772	0.440	0.005			
Age	10.932	<0.001	0.177			
Sex	−2.323	0.020	−0.142			
Pregnancy (girls)^b^				71.684	4	<0.001
*n* = 507, yes = 84 (16.57%)						
LL count	1.099	0.272	0.040			
IQ	−1.953	0.051	−0.019			
SES	−3.209	0.001	−0.103			
Age	6.952	<0.001	0.521			
Pregnancy (boys)^b^				36.727	4	<0.001
*n* = 513, yes = 37 (7.21%)						
LL count	0.666	0.506	0.031			
IQ	5.319	<0.001	0.590			
SES	−1.776	0.076	−0.076			
Age	0.242	0.809	0.003			
Criminal conviction^b^				32.979	5	<0.001
*n* = 1309, yes = 35 (2.67%)						
LL count	−0.418	0.676	−0.020			
IQ	−0.232	0.817	−0.003			
SES	−2.635	0.008	−0.114			
Age	2.775	0.006	0.268			
Sex	−3.845	<0.001	−1.890			
BMI^a^				10.24	5	<0.001
*n* = 1252, M (sd) = 24.08 (5.91), range = 13.83–46.48						
LL count	−0.971	0.332	−0.043			
IQ	−2.133	0.033	−0.024			
SES	1.260	0.208	0.047			
Age	5.937	<0.001	0.517			
Sex	2.771	0.006	0.915			

*Note*: Life outcomes included academic performance (DAWBA parent report), alcohol use frequency (last 12 months), pregnancy (ever becoming pregnant or responsible for a partner's pregnancy ‐ separately for girls and boys), criminal conviction (ever convicted for a crime) and body mass index (BMI).

^a^ Linear regression.

^b^ Logistic regression.

^c^ Number of trials children chose a larger later reward.

Higher IQ and female sex were predictive of better academic performance. Age was associated with increased alcohol use and a higher BMI. Age was also associated with increased likelihood of pregnancy in girls and being responsible for pregnancy in boys, while SES was associated with this outcome only in girls. Male sex was associated with a greater likelihood of criminal conviction.

## DISCUSSION

The current study examined preference for larger delayed rewards versus smaller immediate rewards measured with the CDT in a large, well‐defined community sample of Brazilian children with and without ADHD. It also evaluated longitudinal associations of baseline preference for larger later rewards with psychopathology and life outcomes. Contrary to our expectations, this preference did not differ according to children's ADHD diagnostic status, nor did it predict any psychopathology or functional life outcomes (academic performance, alcohol use, criminal conviction, early pregnancy, or BMI). To our knowledge, this is the first study to examine such longitudinal associations of childhood CDT performance.

The absence of a significant difference in CDT performance between the ADHD and TD group is surprising. A stronger preference for immediate reward has been widely reported in ADHD across experimental paradigms (Furukawa et al., 2022; Sonuga‐Barke et al., 1992; Tripp & Alsop, 2001) with several meta‐analyses making convincing arguments for the stability of such results (Jackson & MacKillop, 2016; Patros et al., 2016; Pauli‐Pott & Becker, 2015). However, the most recent meta‐analysis, which aimed to address methodological concerns of previous studies, identified clear evidence of publication bias in the results of SCP studies (most of which used CDT) (Jackson & MacKillop, 2016; Marx et al., 2021), raising questions about unpublished negative findings (De Vries et al., 2018; Franco et al., 2014; Ioannidis, 2005). The current results are nevertheless consistent with negative CDT findings from other large community sample studies (Bidwell et al., 2007; Wåhlstedt et al., 2009).

Most published SCP studies were conducted in higher‐income, North American/European countries, with the ADHD group typically recruited in clinic settings. While differential CDT performance in individuals with ADHD, compared to typically developing peers, may not be exclusive to these clinical samples, they likely represent more homogeneous and impaired populations. The typically developing groups in these studies are also likely more homogeneous. Such participant characteristics may have contributed to significant group differences. The negative findings in our and other community sample studies (Bidwell et al., 2007; Wåhlstedt et al., 2009) would seem to support this hypothesis. Population differences in preference for immediacy may have also contributed to our findings with Brazilian children from lower‐income families. People in lower‐income countries (Ho et al., 2022; Ruggeri et al., 2022), and children and adolescents in lower income households (DeRosa et al., 2023), have been shown to discount delayed reward more. Typically developing children in Brazil may prefer smaller sooner rewards, reducing the difference with the ADHD group.

Given the assumed links between impulsivity and poor psychiatric and functional outcomes (Dick et al., 2010; Loeber et al., 2012; Whiteside & Lynam, 2003), we were surprised there were no significant associations between the preference for the larger later rewards and risk for longer‐term psychiatric disorders or functional outcomes. However, longitudinal associations of such preference or the ability to choose delayed ‘better’ rewards are not well characterized. Despite the popularity of the SCP, no other published longitudinal data were identified. The current results are consistent with recent research questioning the predictive validity of these constructs, at least as measured by existing experimental tasks (Ho et al., 2022; Isen et al., 2014; Michaelson & Munakata, 2020; Watts et al., 2018).

Consistent with previous research (Albert, 2015; Murray et al., 2013; Wildeman & Wang, 2017), sex and SES were associated with longitudinal outcomes. Lower SES was predictive of greater likelihood of pregnancy in girls. Females were more likely to meet diagnostic criteria for a psychiatric disorder, while males were more likely to be convicted of a criminal offence. A similar pattern of findings was reported in a reanalysis of early delay of gratification findings with the marshmallow task. Michaelson and Munakata (2020) reported that children's social environment but not marshmallow task performance predicted later functioning. Together, these findings highlight the importance of social factors as predictors of later functioning.

The current study has a number of strengths. The sample is large, well defined and diverse. Importantly, the study tests the predictive power of the CDT task for longitudinal psychiatric and functional outcomes. On the other hand, the number of CDT trials is smaller (15 vs. 20) and the length of the wait for the larger reward is shorter (20 s vs. 30 s) than in many SCP studies, which may have impacted the findings. That said, the standard deviations in our sample are comparable to other CDT studies (see Marx et al., 2021). Participants knew they would receive candies as rewards after the task, which may have served to reduce group differences. Previously reported group differences are smaller when real rather than hypothetical rewards are used (Marx et al., 2021). The severity of participants' psychiatric morbidity in our community sample may be lower, compared to the clinical samples used in other studies, although still meeting the DSM criteria. The diagnoses at the baseline and 3‐year follow up were based on clinical interviews with caregivers for all participants, while at the 6‐year follow up, clinical interviews were conducted with participants themselves if they were 18 or older (728 [52.53%]). This, together participants being older, likely contributed to the reduction in the prevalence rates of externalizing disorders, including ADHD, overtime. Some participants dropped out of the study over the course of 6 years; however, the impact of psychiatric diagnoses or CDT performance (Supplemental Table S6 and S7) on the dropouts were small, and therefore these dropouts are unlikely to be the reason for the observed results. The CDT was administered only at baseline data collection. As the ability to control impulsive choices appears to fluctuate during childhood and adolescence (Klein et al., 2022), it would be important to examine the relationship between changes in impulsive choice (DeRosa et al., 2023) and functional outcomes. Longitudinal follow‐up extended to 6 years post baseline and some participants were still adolescents; the longer‐term effects on life outcomes should be explored in future studies. Finally, we urge caution in generalizing from the current findings of no association between CDT performance in childhood and later functional impairments. We recommend testing the longitudinal association between impulsive choices and such impairments using a range of experimental tasks and other approaches, for example, questionnaires and ecological momentary assessments.

In summary, while previous research has reported differences in the preference for larger delayed rewards in children with and without ADHD, this was not replicated in the current study with a large community sample of Brazilian children. Further, there is no evidence from the current analyses to suggest that childhood performance on the CDT predicts longer‐term life outcomes. The findings have important implications for the field. The SCP may not be as sensitive to ADHD versus TD group difference as assumed. Expanding methodology to evaluate waiting behavior and impulsive choices, as well as sample diversity, would extend understanding of the nature of altered reward sensitivity in ADHD. While it has been widely assumed that not waiting for larger rewards is associated with negative outcomes, the current results, together with a careful review of the wider literature, offer little evidence for such effects. The potential impact of publication and reporting bias on knowledge accumulation should be considered, and additional, and more ecologically valid, studies may help clarify these concerns.

## AUTHOR CONTRIBUTIONS

**Patricia P. Bado**: Conceptualization; data curation; formal analysis; investigation; methodology; writing – original draft; writing – review & editing. **Giovanni A. Salum**: Funding acquisition; methodology; project administration; supervision; writing – review & editing. **Luis A. Rohde**: Funding acquisition; writing – review & editing. **Ary Gadelha**: Writing – review & editing. **Pedro M. Pan**: Funding acquisition; writing – review & editing. **Eurípedes C. Miguel**: Funding acquisition; project administration; resources; writing – review & editing. **Gail Tripp**: methodology; writing – review & editing. **Emi Furukawa**: Conceptualization; data curation; formal analysis; investigation; project administration; supervision; writing – original draft; writing – review & editing.

## CONFLICT OF INTEREST STATEMENT

The authors have declared they have no competing or potential conflicts of interest.

## ETHICAL CONSIDERATIONS

This study was approved by the ethics committees of the University of São Paulo, the Federal University of Rio Grande do Sul, and other local institutions that took part in the data collection. All participants and their caregivers provided informed written consent.

## Supporting information

Supporting Information S1

## Data Availability

Data is available upon request.

## ENDNOTES

^1^
The task included 15 trials rather than the 20 trials, and the delay for LL was 20s rather than 30s, used in many studies, due to time constraints. This decision was made in consultation with the original task developer (Sonuga‐Barke et al., 1992). The standard deviations of the LL% (21–23%) were comparable with CDT studies using the 20 trial/30s version of the task (Marx et al., 2021).

^2^
While the TD group sample size was significantly larger than the ADHD group sample size, the error variances of the two groups were not significantly different. When means were generated for 10 subsamples of TD participants, drawn at random to match the ADHD sample size (*n* = 213), the TD group LL mean ranged from 5.56 to 6.53.

^3^
Post‐hoc analyses demonstrated no significant effect of sex on the LL count for the TD or ADHD group, and no significant TD versus ADHD group differences on the LL count for girls or boys.
