# Supplementary material for: Is waiting for rewards good for you? No association between impulsive choice, psychopathology, and functional outcomes in a large cohort sample
Source: JCPP Adv. 2024 Apr 15;4(2):e12231. doi: 10.1002/jcv2.12231 (PMC11143955; doi:10.1002/jcv2.12231)
Supplement: Supplementary file 1 — Supporting Information S1 [file JCV2-4-e12231-s001.docx]

| Supplemental Table 1. Regression models with LL predicting current and future psychopathology. | | | | | | |
| --- | --- | --- | --- | --- | --- | --- |
|  |  |  |  |  |  |  |
| **Baseline** |  |  |  |  |  |  |
|  | **z** | **p** | **B** | **Chi-square** | **df** | **p** |
| **ADHD** |  |  |  |  |  |  |
| LL count | 1.925 | .054 | .037 | 3.660 | 1 | .056 |
| **Behavioral** |  |  |  |  |  |  |
| LL count | -.418 | .676 | -.010 | .175 | 1 | .675 |
| **Emotional** |  |  |  |  |  |  |
| LL count | -.008 | .994 | .000 | .000 | 1 | .994 |
| **Any disorder** |  |  |  |  |  |  |
| LL count | .963 | .335 | .013 | .925 | 1 | .336 |
|  |  |  |  |  |  |  |
| **3-year follow-up** | |  |  |  |  |  |
| **ADHD** |  |  |  |  |  |  |
| LL count | -.091 | .928 | -.003 | .008 | 1 | .928 |
| **Behavioral** |  |  |  |  |  |  |
| LL count | -.194 | .846 | -.006 | .038 | 1 | .846 |
| **Emotional** |  |  |  |  |  |  |
| LL count | -.215 | .829 | -.004 | .047 | 1 | .829 |
| **Any disorder** |  |  |  |  |  |  |
| LL count | .003 | .998 | .000 | .000 | 1 | .998 |
|  |  |  |  |  |  |  |
| **6-year follow-up** | |  |  |  |  |  |
| **ADHD** |  |  |  |  |  |  |
| LL count | -.055 | .956 | -.002 | .003 | 1 | .956 |
| **Behavioral** |  |  |  |  |  |  |
| LL count | .360 | .719 | .014 | .129 | 1 | .720 |
| **Emotional** |  |  |  |  |  |  |
| LL count | .213 | .831 | .004 | .045 | 1 | .831 |
| **Any disorder** |  |  |  |  |  |  |
| LL count | -.479 | .632 | -.008 | .230 | 1 | .632 |

Supplemental Table 2. CDT performance by three age groups.

|  |  |  |  |
| --- | --- | --- | --- |
|  | **6-8 year-olds** | **9-11 year-olds** | **12-14 year-olds** |
| **TD** |  |  |  |
| n | 429 | 651 | 299 |
| LL count M (sd) | 5.80 (3.70) | 5.89 (3.67) | 6.23 (3.70) |
|  |  |  |  |
| **ADHD** |  |  |  |
| n | 64 | 116 | 33 |
| LL count M (sd) | 5.77 (3.75) | 6.72 (4.03) | 6.76 (3.41) |

Supplemental Table 3. CDT performance by gender.

|  | **girls** | **boys** |
| --- | --- | --- |
| **TD** |  |  |
| n | 635 | 744 |
| LL count M (sd) | 5.85 (3.60) | 6.02 (3.76) |
|  |  |  |
| **ADHD** |  |  |
| n | 83 | 130 |
| LL count M (sd) | 6.47 (3.88) | 6.42 (3.87) |

Supplemental Table 4. SDQ hyperactivity/inattention scale scores.

|  | **girls** | **boys** |
| --- | --- | --- |
| **TD** |  |  |
| n | 635 | 744 |
| SDQ M (sd) | 3.86 (2.88) | 4.35 (2.79) |
|  |  |  |
| **ADHD** |  |  |
| n | 83 | 130 |
| SDQ M (sd) | 8.23 (1.73) | 8.25 (1.88) |

Figure S1. Effect sizes for associations between sex and risk for psychopathology. Odds ratios and 95% confidence intervals for children meeting diagnostic criteria versus not according to their sex. Values greater than one indicate increased risks for diagnosis in girls. 3y: 3-year follow-up; 6y: 6-year follow-up.


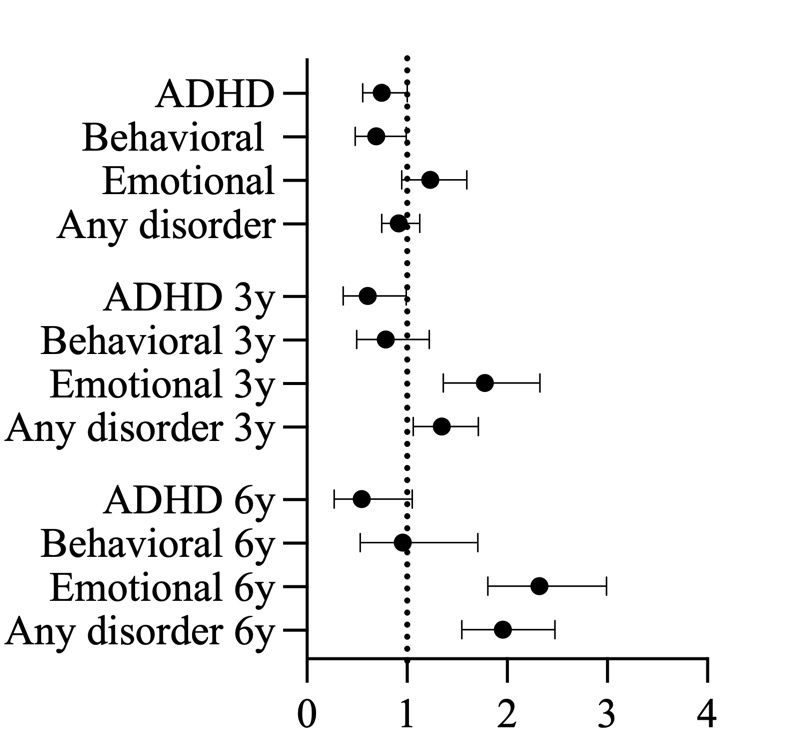


Supplemental Table 5. Odds ratios and 95% confidence intervals for children meeting diagnostic criteria versus not. Values greater than one indicate increased risks for psychopathology.

| **Baseline** | | | |  | **3-year follow-up** | | |  | **6-year follow-up** | | |
| --- | --- | --- | --- | --- | --- | --- | --- | --- | --- | --- | --- |
| **ADHD** | **OR** | **2.5%** | **97.5%** |  | **OR** | **2.5%** | **97.5%** |  | **OR** | **2.5%** | **97.5%** |
| LL count | 1.042 | 1.002 | 1.082 |  | 1.000 | .937 | 1.065 |  | 1.001 | .916 | 1.089 |
| IQ | .982 | .972 | .993 |  | .982 | .965 | .998 |  | .986 | .963 | 1.008 |
| SES | .990 | .958 | 1.023 |  | 1.007 | .953 | 1.062 |  | .951 | .880 | 1.025 |
| Age | .956 | .886 | 1.031 |  | .869 | .763 | .988 |  | .941 | .793 | 1.112 |
| Sex | .748 | .557 | 1.001 |  | .608 | .363 | .994 |  | .548 | .271 | 1.054 |
| **Behavioral** |  |  |  |  |  |  |  |  |  |  |  |
| LL count | .991 | .944 | 1.040 |  | .995 | .936 | 1.054 |  | 1.014 | .936 | 1.095 |
| IQ | .992 | .979 | 1.004 |  | .993 | .977 | 1.008 |  | .988 | .967 | 1.008 |
| SES | .943 | .905 | .983 |  | .955 | .906 | 1.006 |  | .974 | .908 | 1.041 |
| Age | .990 | .902 | 1.086 |  | 1.056 | .941 | 1.185 |  | 1.118 | .961 | 1.303 |
| Sex | .694 | .481 | .993 |  | .787 | .500 | 1.223 |  | .956 | .531 | 1.706 |
| **Emotional** |  |  |  |  |  |  |  |  |  |  |  |
| LL count | 1.001 | .966 | 1.037 |  | .998 | .962 | 1.034 |  | 1.004 | .971 | 1.039 |
| IQ | .992 | .983 | 1.001 |  | .990 | .980 | .999 |  | 1.010 | 1.001 | 1.018 |
| SES | .981 | .952 | 1.010 |  | 1.001 | .971 | 1.032 |  | .993 | .965 | 1.022 |
| Age | 1.041 | .973 | 1.114 |  | 1.053 | .983 | 1.129 |  | 1.021 | .957 | 1.090 |
| Sex | 1.231 | .949 | 1.596 |  | 1.780 | 1.363 | 2.328 |  | 2.324 | 1.809 | 2.995 |
| **Any disorder** |  |  |  |  |  |  |  |  |  |  |  |
| LL count | 1.015 | .988 | 1.044 |  | 1.002 | .970 | 1.034 |  | .993 | .961 | 1.025 |
| IQ | .990 | .982 | .997 |  | .990 | .982 | .998 |  | 1.007 | .999 | 1.016 |
| SES | .980 | .957 | 1.003 |  | .992 | .965 | 1.020 |  | .993 | .967 | 1.020 |
| Age | 1.000 | .948 | 1.055 |  | 1.038 | .975 | 1.104 |  | 1.016 | .956 | 1.080 |
| Sex | .918 | .749 | 1.126 |  | 1.349 | 1.064 | 1.710 |  | 1.956 | 1.546 | 2.479 |

Supplemental Table 6. Contributions of the LL performance and covariate variables to the likelihood of remaining in the study (all participants).

| 3-year follow-up | |  |  |  |  |  |
| --- | --- | --- | --- | --- | --- | --- |
|  | **z** | **p** | **B** | **Chi-square** | **df** | **p** |
| **Participation status*** | |  |  | 19.965 | 5 | .001 |
| LL count | 1.840 | .066 | .031 |  |  |  |
| IQ | 2.270 | .023 | .010 |  |  |  |
| SES | 1.032 | .302 | .014 |  |  |  |
| Age | -2.535 | .011 | -.079 |  |  |  |
| Sex | 1.550 | .121 | .185 |  |  |  |

*0 = participants who dropped out (n = 348, M = 5.65, SD = 3.46), 1 = participants who remained in the study (n = 1569, M = 6.05, SD = 3.72). Positive Beta value for the LL count means that participants who are more likely to choose LL rewards are more likely to stay in the study.

| 6-year follow-up | |  |  |  |  |  |
| --- | --- | --- | --- | --- | --- | --- |
|  | **z** | **p** | **B** | **Chi-square** | **df** | **p** |
| **Participation status*** | |  |  | 6.018 | 5 | .304 |
| LL count | 1.601 | .109 | .023 |  |  |  |
| IQ | .500 | .617 | .002 |  |  |  |
| SES | 1.038 | .299 | .012 |  |  |  |
| Age | -1.054 | .292 | -.028 |  |  |  |
| Sex | -1.085 | .278 | -.112 |  |  |  |

*0 = participants who dropped out (n = 531, M = 5.77, SD = 3.59), 1 = participants who remained in the study (n = 1386, M = 6.06, SD = 3.71). Positive Beta value for the LL count means that participants who are more likely to choose LL rewards are more likely to stay in the study.

Supplemental Table 7. Contributions of the LL performance and covariate variables to the likelihood of remaining in the study (ADHD participants).

| 3-year follow-up | |  |  |  |  |  |
| --- | --- | --- | --- | --- | --- | --- |
|  | **z** | **p** | **B** | **Chi-square** | **df** | **p** |
| **Participation Status*** | |  |  | 8.158 | 5 | .148 |
| LL count | -1.031 | .303 | -.053 |  |  |  |
| IQ | -1.246 | .213 | -.018 |  |  |  |
| SES | 1.997 | .046 | .110 |  |  |  |
| Age | -.614 | .539 | -.073 |  |  |  |
| Sex | 1.304 | .192 | .527 |  |  |  |

*0 = participants who dropped out (n = 30, M = 7.17, SD = 3.77), 1 = participants who remained in the study (n = 183, M = 6.32, SD = 3.88). Negative Beta value for the LL count means that participants who are more likely to choose LL rewards are less likely to stay in the study.

| 6-year follow-up | |  |  |  |  |  |
| --- | --- | --- | --- | --- | --- | --- |
|  | **z** | **p** | **B** | **Chi-square** | **df** | **p** |
| **Participation Status*** | |  |  | 5.394 | 5 | .370 |
| LL count | -2.003 | .045 | -.084 |  |  |  |
| IQ | .016 | .987 | .000 |  |  |  |
| SES | .881 | .379 | .037 |  |  |  |
| Age | -.475 | .635 | -.046 |  |  |  |
| Sex | -.556 | .578 | -.190 |  |  |  |

*0 = participants who dropped out (n = 50, M = 7.42, SD = 3.86), 1 = participants who remained in the study (n = 163, M = 6.13, SD = 3.83). Negative Beta value for the LL count means that participants who are more likely to choose LL rewards are less likely to stay in the study.
